# Supplementary material for: Paraoxonase 1 Phenotype and Protein N-Homocysteinylation in Patients with Rheumatoid Arthritis: Implications for Cardiovascular Disease
Source: Antioxidants (Basel). 2020 Sep 21;9(9):899. doi: 10.3390/antiox9090899 (PMC7555791; doi:10.3390/antiox9090899)
Supplement: Supplementary file 1 [file antioxidants-09-00899-s001.pdf]

Table S1. PON1 polymorphism

**Phenotype (genotype)**	**Control (n=70)**	**RA (n=74)**
QQ	37	39
QR	31	29
RR	2	6

Table S2. Specific PON1 activities.

	**Control (n=70)**	**RA (n=74)**
Phenyl acetate (U/ μg)	4.71 ± 1.04	3.41 ± 0.62*
Paraoxon (U/μg)		
QQ	5.72 ± 0.56	3.52 ± 0.47**
QR	7.85 ± 0.84	5.82 ± 1.03**
RR	10.2 ± 0.66	9.18 ± 0.53
Hcy thiolactone (nmol × min^{-1} × μg^{-1})	3.28 ± 0.66	2.37 ± 0,43**

*p<0.05, **p<0.01 vs. control group

Table S3. PON1 status, Hcy and Hcy thiolactone in healthy subjects and RA patients eGFR>90 ml/min).

	**Control (n=54)**	**RA (n=50)**
PON1 polymorphism		
QQ	29	27
QR	23	20
RR	2	3
PON1 concentration (μg/ml)	22.4 ± 3.1	20.6 ± 2.3
PON1 activity toward phenyl acetate (U/ml)	122.3 ± 15.1	73.5 ± 12.3**
PON1 activity toward Hcy thiolactone (nmol × min^{-1} × ml^{-1})	84.7 ± 11.2	50.6 ± 9.6***
Total homocysteine (μM)	7.98 ± 1.01	8.26 ± 1.3
N-Hcy-protein (μM)	2.24 ± 0.82	2.66 ± 0.96*

*p<0.05, **p<0.01, ***p<0.001 vs. control group

Table S4. Serum lipids, PON1 status, Hcy and N-Hcy protein in healthy females according to menopausal status

	**Before menopause (n=10)**	**After menopause (n=48)**
Age	43.4 ± 8.56	58.8 ± 6.11***
Total cholesterol (mg/dl)	184.0 ± 29.4	220.4 ± 30.0*
LDL-cholesterol (mg/dl)	108.6 ± 17.4	129.2 ± 29.0
HDL-cholesterol (mg/dl)	56.4 ± 12.5	69.4 ± 15.8
Non-HDL cholesterol (mg/dl)	127.6 ± 19.7	150.9 ± 30.9
Triglycerides (mg/dl)	94.4 ± 35.3	108.7 ± 39.3
PON1 concentration (μg/ml)	20.7 ± 3.2	21.6 ± 2.9
PON1 activity toward Hcy thiolactone (nmol × min^{-1} × ml^{-1})	89.7 ± 11.6	85.4 ± 10.9
Total homocysteine (μM)	7.11 ± 1.12	7.44 ± 1.07
N-Hcy-protein (μM)	2.24 ± 0.82	2.66 ± 0.96

*p<0.05, ***p<0.001 vs. premenopausal group

Table S5. Serum lipids, disease activity, PON1 status, Hcy and N-Hcy protein in females with RA according to menopausal status

	**Before menopause (n=21)**	**After menopause (n=39)**
Age	39.4 ± 8.27	60.3 ± 7.18***
Total cholesterol (mg/dl)	197.8 ± 53.8	185.5 ± 56.9
LDL-cholesterol (mg/dl)	111.5 ± 36.0	105.7 ±31.9
HDL-cholesterol (mg/dl)	66.3 ± 17.2	56.9 ± 18.2
Non-HDL cholesterol (mg/dl)	131.4 ± 52.2	128.6 ± 35.0
Triglycerides (mg/dl)	100.0 ± 48.9	114.7 ± 48.1
ESR (mm/1 h)	29.0 ± 19.4	41.3 ± 26.5
CRP (mg/l)	13.4 ± 13.7	37.4 ± 37.8**
DAS28-ESR	4.67 ± 1.36	5.51 ± 1.88
PON1 concentration (μg/ml)	23.7 ± 3.0	21.8 ± 2.9
PON1 activity toward Hcy thiolactone (nmol × min^{-1} × ml^{-1})	54.6 ± 8.2	50.5 ± 8.6
Total homocysteine (μM)	7.25 ± 1.00	8.56 ± 1.2*
N-Hcy-protein (μM)	2.59 ± 0.78	2.73 ± 0.69

*p<0.05, **p<0.01, ***p<0.001 vs. premenopausal group
